# Human plasma inositol hexakisphosphate (InsP _6_ ) phosphatase identified as the Multiple Inositol Polyphosphate Phosphatase 1 (MINPP1)

**DOI:** 10.17912/micropub.biology.001390

**Published:** 2024-11-01

**Authors:** Valeria Fedeli, Jingyi Wang, Vincent Cantagrel, Adolfo Saiardi

**Affiliations:** 1 Laboratory for Molecular Cell Biology, London WC1E 6BT, UK, University College London, London, England, United Kingdom; 2 Developmental Brain Disorders Laboratory, Université Paris Cité, INSERM UMR1163, Imagine Institute, 75015, Paris, France, Université Paris Cité, Paris, Île-de-France, France

## Abstract

Inositol hexakisphosphate (InsP _6_ ), also known as phytic acid, is a potent chelator of bivalent cations. Intracellular InsP _6_ molecules are associated with magnesium. Calcium is the prevalent bivalent cation outside the cell and its association with InsP _6_ could lead to the formation of insoluble complexes. To avoid the formation of dangerous InsP _6_ /Calcium precipitates in the bloodstream, mammals must possess a robust InsP _6_ phosphatase in their plasma. Here we identify the Multiple Inositol Polyphosphate Phosphatase 1 ( MINPP1 ) as the InsP _6_ phosphatase present in human plasma.

**Figure 1. Identification of Minpp1 as the plasma InsP 6 phosphatase preventing InsP 6 precipitation.**
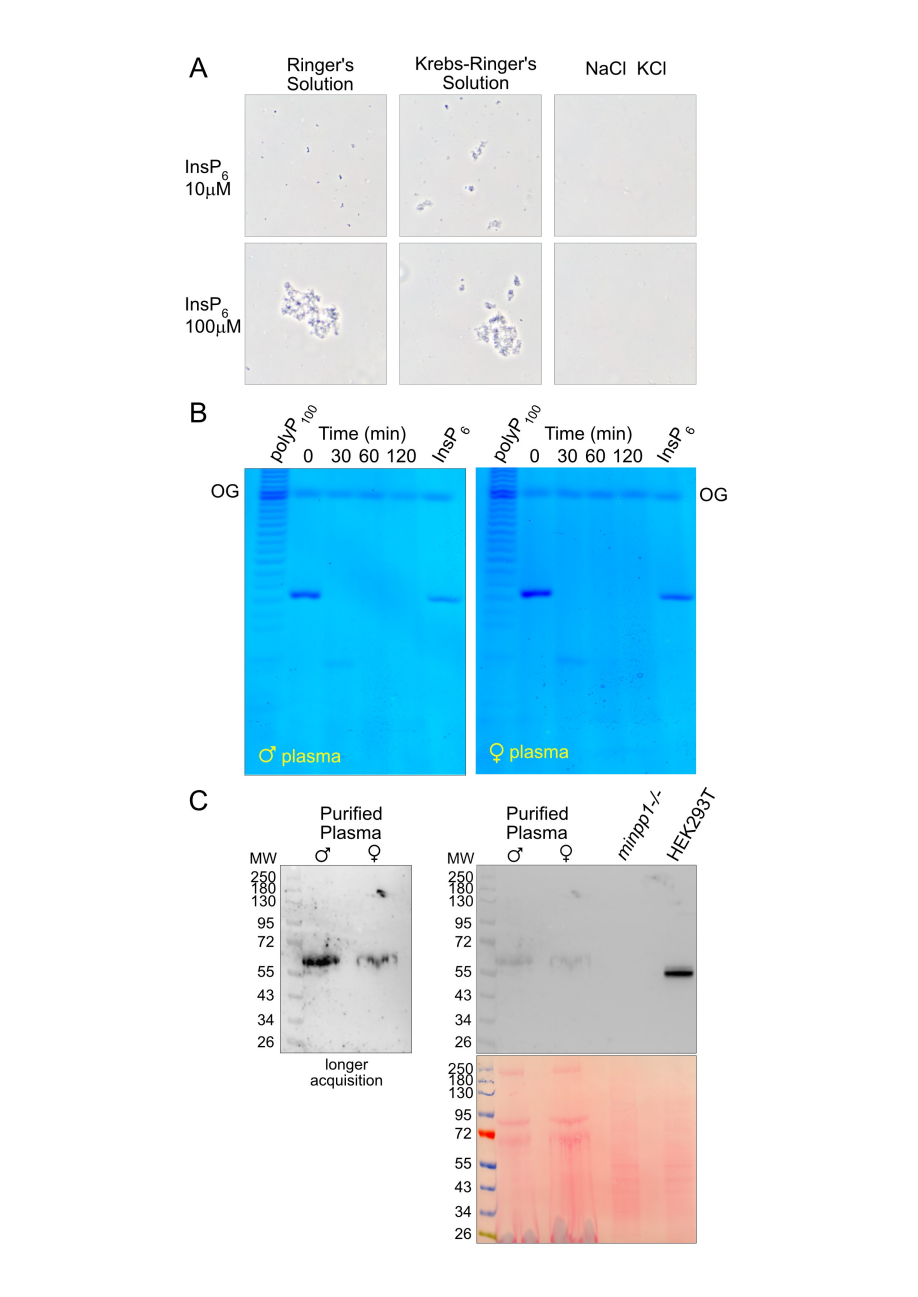
A) The indicated amount of InsP _6_ was added to 1 ml of Ringer's solution (147 mM NaCl, 4 mM KCl, 3 mM CaCl _2_ ) Krebs-Ringer's solution (120 mM NaCl, 5 mM KCl, 2 mM CaCl2, 1 mM MgCl2, 25 mM NaHCO3) and control salt solution NaCl KCl (147 mM NaCl, 4 mM KCl) and incubated at 37 ^o^ C for 30 minutes with rotation. InsP _6_ -Ca precipitated were observed by phase contrast microscopy. B) 5 nmol of InsP _6_ was added to 1 ml of human plasma and incubated at 37°C for the indicated time before acidification and extraction of inositol phosphates with the TiO _2_ procedure (Wilson & Saiardi, 2018). The extracts were loaded on 33% polyacrylamide gel and stained with toluidine blue (Lonetti et al., 2011). To orientate the gel polyP100 (50 nmol [Pi]) was loaded on the left and InsP _6_ (2 nmol) was used as migration standard. OrangeG (OG) dye was used to monitor gel electrophoresis. C) Albumin and immunoglobulin-depleted male and female plasma (50 mg) were used to perform Western blot with anti Minpp1 antibody. HEK293T and isogenic minpp1-/- extracts (10 mg) (Ucuncu et al., 2020) were used as positive and negative controls respectively. Shown are the representative results of at least three biological repeats.

## Description

Inositol phosphates (InsPs) represent a diverse and important class of intracellular signalling molecules (for review see (Kim et al., 2024) ). The fully phosphorylated inositol ring of inositol hexakisphosphate (InsP _6_ ) represents the most abundant InsPs present in mammalian cells, with intracellular concentrations ranging from 20 to 100 µM (Qiu et al., 2020; Shears, 2001) . InsP _6_ not only acts as a structural component of proteins such as the HIV capsid (Mallery et al., 2019) and the RNA-editing deaminase ADAR2 (Macbeth et al., 2005) , but also regulates signalling pathways such as necroptosis by activating mixed lineage kinase domain-like ( MLKL ) protein (Dovey et al., 2018) and protein phosphorylation by activating casein kinase 2 (CK2) (Solyakov et al., 2004) . Additionally, InsP _6_ is the main precursor of inositol pyrophosphates such as InsP _7_ and InsP _8_ , which are important signalling molecules themselves (Nguyen Trung, et al., 2022; Wilson et al., 2013) . The charged nature of InsPs prevents their diffusion across the plasma membrane, and therefore, InsP's metabolic and signalling networks are exclusively intracellular with many InsPs-kinases localized within the cytosol or into the nucleus (Otto et al., 2007; Shears, 2004) . We could envisage, however, that cell death mechanisms and subsequent cell lysis could lead to the release of intracellular InsPs to our bloodstream. What is the fate of this theoretical extracellular pool of InsPs? Here we focus our attention on InsP _6_ .

Inositol hexakisphosphate possesses a unique charge density, with its twelve negative charges constrained around the carbon backbone of inositol. The biophysical properties of InsP _6_ and its association with bivalent cations have been intensively studied (Hager et al., 2016; Kurz et al., 2023) . Seminal thermal analysis and solubility measurements studies have determined that in the cytosolic environment, in which magnesium is the prevalent bivalent cation (Romani, 2011) , InsP _6_ could exist as a soluble penta-magnesium complex up to a concentration of 49 µM (Torres et al., 2005; Veiga et al., 2006) . To verify InsP _6_ solubility in conditions mimicking the salt composition of the human plasma, we took advantage of two intravenous fluids used to treat dehydration: the glucose-depleted Ringer's solution (147 mM NaCl, 4 mM KCl, 3 mM CaCl _2_ ) and the glucose-depleted Krebs-Ringer's solution (120 mM NaCl, 5 mM KCl, 2 mM CaCl _2_ , 1 mM MgCl _2_ , 25 mM NaHCO _3_ ) and as a control the same mix of sodium and potassium salts (147 mM NaCl, 4 mM KCl) omitting bivalent cations. Using phase contrast microscopy, we observed the formation of insoluble precipitates when physiological levels of InsP _6_ (10 µM and 100 µM) were added to Ringer's or Krebs-Ringer's solution, in a calcium-dependent manner ( Figure 1A ). Hence, these precipitates must be InsP _6_ /Calcium complexes. This qualitative visual result is consistent with the quantitative measurements reporting the insolubility of InsP _6_ in solutions containing calcium (Veiga et al., 2006) , which is the most prevalent bivalent cation present in plasma (Allgrove, 2009) . The prompt precipitation of InsP _6_ /Calcium complexes in plasma-mimicking solutions suggests that mammals must have evolved mechanisms to cleanse InsP _6_ from plasma, to avoid the formation of harmful precipitates in their circulatory system. Indeed, we previously demonstrated the presence of a robust InsP _6_ dephosphorylation activity in mammalian plasma (Irvine et al., 2015; Wilson et al., 2015) . Here we repeated the InsP _6_ dephosphorylation assay using human plasma samples from both male and female donors. The incubation of InsP _6_ (5 µM) in plasma from both genders at 37 ^o^ C resulted in InsP _6_ dephosphorylation over time ( Figure 1B ), thus confirming previous findings (Irvine et al., 2015; Wilson et al., 2015) .

The human genome carries a single gene encoding a phosphatase active towards InsP _6_ , namely Multiple Inositol Polyphosphate Phosphatase 1 ( MINPP1 ). This enzyme belongs to a conserved family of histidine acid phosphatases (IPR016274), commonly referred to as phytases because acting on phytic acid another name for InsP _6_ . The thoughtful characterization of MINPP1 enzymatic activities *in vitro* and *in vivo* revealed that this enzyme dephosphorylates InsP _6_ primarily to Ins(1,2)P _2_ (Nguyen Trung, Kieninger, et al., 2022) . By regulating InsP _6_ metabolism, Minnp1 controls numerous cellular processes; additionally, recent Mendelian genetic studies have revealed the importance of MINPP1 in the pathophysiology of a specific form of pontocerebellar hypoplasia (PCH) (Appelhof et al., 2021; Ucuncu et al., 2020) , a severe neurodegenerative disorder. To verify whether MINPP1 is present in human plasma to account for the observed InsP _6_ phosphatase activity ( Figure 1B ), we utilized an immunochemical assay. To apply this approach to plasma, it is crucial to remove the albumin and immunoglobulins (mainly IgG), which represent about 60% and 20% of the proteome in the plasma, respectively and could interfere with immunoblotting. Using a commercially available albumin and IgG depletion kit we enriched plasma proteome. The western blot performed on the eluate enriched for non-albumin and non-IgG proteins employing anti MINPP1 antibody demonstrates the presence of this critical InsP _6_ phosphatase in human plasma ( Figure 1C ). Our direct analysis confirms a mass spectrometry (MS) based study identifying Minpp1 in human plasma (Farrah et al., 2011) . Different sample preparation approaches, MS techniques, and algorithms used to extract MS data could lead to the identification of different sets of proteins from human plasma. In fact, a recent meta-analysis aimed at generating a reference set of plasma proteome to be used for targeted MS does not include MINPP1 (Kliuchnikova et al., 2023) . Nevertheless, MINPP1 is one of the 4072 plasma proteins listed by the Human Protein Atlas ( https://www.proteinatlas.org ), here Minpp1 presence is recorded as non-validated by blood-based immunoassay. Our study unequivocally provides this important evidence.

Our confirmation of MINPP1 in human plasma should put to rest the debate on the presence of InsP _6_ in plasma (Irvine, 2014; Irvine et al., 2015) that few authors have been able to detect using obviously unreliable analytical methods. The presence of Minpp1 in plasma deemed it unlikely for plasma to contain InsP _6_ . Our finding also prompts a revaluation of the literature that suggest a direct health-beneficial role of orally administrated InsP _6_ . Any positive dietary benefit of InsP _6_ is likely to have derived from its catabolism to propionate by gut bacteria (De Vos et al., 2024) or from its dephosphorylation to inositol in the gut, which is subsequently absorbed by the intestine.

MINPP1 is predominantly localized inside the endoplasmic reticulum (ER) since it possesses an ER retention signal (KDEL) (Kilaparty et al., 2014; Yu et al., 2023) . Secretory vesicles may emerge from ER-Golgi vesicular trafficking pathways which ultimately facilitate the release of MINPP1 into the plasma. Since secretion of KDEL protein has been reported (Palazzo et al., 2022) , further studies aimed at characterizing the secretory mechanisms of MINPP1 should be performed to fully appreciate the physiological functions of this important InsP _6_ phosphatase outside the cell.

The demonstration of extracellular MINPP1 opens new perspectives to interpret the role of this InsP _6_ phosphatase might play in disease conditions (Appelhof et al., 2021; Ucuncu et al., 2020) . In the absence of MINPP1 , neural cell death could be associated with a release of InsP _6_ , leading to InsP _6_ /Calcium precipitates in the extracellular space, potentially contributing to pathogenicity. Pathology mechanism could result from a "vicious circle" combining both intra and extracellular deleterious consequences on neural cell differentiation and survival during brain development. Our result raises the possibility of MINPP1 's presence in the cerebrospinal fluid (CSF). This is a tantalising prospect that warrant the urgent need for further studies in the physiology of MINPP1 .

## Methods

InsP _6_ solubility study.

To study the solubility of InsP _6_ , we used three different solutions: glucose-depleted Ringer's solution (147 mM NaCl, 4 mM KCl, 3 mM CaCl _2_ ), glucose-depleted Krebs-Ringer's solution (120 mM NaCl, 5 mM KCl, 2 mM CaCl _2_ , 1 mM MgCl _2_ , 25 mM NaHCO _3_ ), and a control solution identical to the Ringer's solution but without calcium (147 mM NaCl, 4 mM KCl). InsP _6_ was added to each solution at final concentrations of 10 µM and 100 µM. The mixtures were incubated with rotation at 37°C for 30 minutes. Following incubation, samples were examined under a phase contrast microscope (Olympus CX41) to detect the presence of insoluble InsP _6_ -Ca precipitates.

InsP _6_ dephosphorylation assay.

Male and Female human plasma were bought from TCS Biosciences (Cat: PR200-F-100-H2). Each 1 mL plasma sample was supplemented with a HEPES-MgCl _2_ solution to achieve final concentrations of 2 mM HEPES and 1 mM MgCl _2_ . InsP _6_ (5 µM) (Calbiochem, Sigma-Aldrich, Cat: 407125) was added, and reactions were incubated at 37°C for 20, 60, and 120 minutes. Reactions were stopped by adding 20 µL of a stop solution (100 mM EDTA; 100 mM EGTA). Following incubation, inositol phosphates were purified using the TiO2 method as previously described (Wilson & Saiardi, 2018) and analysed by polyacrylamide gel electrophoresis PAGE followed by toluidine blue staining (Losito et al., 2009) .

Albumin/IgG depletion.

Albumin and IgG were depleted from plasma using the ProteoExtract® Albumin-IgG Removal Kit MAXI (Calbiochem, Sigma-Aldrich, Cat: 122643). Plasma samples were diluted 1:10 in 10X Binding Buffer, and columns were equilibrated with 1X Binding Buffer. The diluted samples were passed through the column, albumin and IgG depleted eluate was collected by washing the column with 2M salt solution as for manufacturer's instructions.

Western Blot Assay.

Plasma samples depleted of IgG and albumin were concentrated and desalted using Amicon® Ultra-0.5 Centrifugal Filter Devices (Millipore, Cat.: UFC501096) according to the manufacturer's protocol. The eluted and concentrated fractions were quantified using the DC™ Protein Assay (Bio-Rad, Reagent A, Cat.: #5000113; Reagent B, Cat.: #5000114; Reagent S, Cat.: #500-0115). Plasma proteins (50 µg) were resolved by electrophoresis using 4-12% Bis-Tris polyacrylamide gels (NuPAGE™, Invitrogen, REF. NP0321BOX) and transferred to nitrocellulose membranes (GE Healthcare Life Sciences Whatman™, Cat.: 10401396). Membranes were blocked in 5% non-fat milk in TBS-T (0.1%) and incubated overnight at 4°C with MINPP1 primary antibody (Santa Cruz, Cat: SC-514214). After three washes in TBS-T (0.1%), membranes were incubated with a secondary anti-mouse IgG1 antibody (Invitrogen, Cat: PA-74421) for 1 hour at room temperature. Detection was performed using the Clarity™ Western ECL substrate (Bio-Rad, Cat.: #170-5060) and images were acquired with the Alliance Q9 imaging system.
